# Ethyl 3-(4-chloro­phen­yl)-2-(dipentyl­amino)-4-oxo-5-phenyl-4,5-dihydro-3*H*-pyrrolo­[3,2-*d*]pyrimidine-7-carboxyl­ate

**DOI:** 10.1107/S1600536812024609

**Published:** 2012-06-02

**Authors:** Ping He, Qin-Qin Wan, Quan-Lei Liao

**Affiliations:** a Research Center for Materials Science & Engineering, Hubei University of Arts and Science, Xiangyang 441053, People’s Republic of China; b College of Chemical Engineering and Food Science, Hubei University of Arts and Science, Xiangyang 441053, People’s Republic of China

## Abstract

In the title compound, C_31_H_37_ClN_4_O_3_, the fused rings of the pyrrolo­[3,2-*d*]pyrimidine system form a dihedral angle of 5.80 (11)°. The phenyl and benzene rings are twisted with respect to the mean plane of the pyrrolo­[3,2-*d*]pyrimidine system [maximum deviation = 0.077 (2) Å], making dihedral angles of 61.05 (12) and 75.39 (10)°, respectively. The eth­oxy group is disordered over two positions with the site-occupancy ratio fixed at 0.54:0.46. In the crystal, mol­ecules are linked *via* C—H⋯O hydrogen bonds, forming a two-dimensional network lying parallel to the *ab* plane. There are also π–π [centroid–centroid distances = 3.5954 (17) and 3.965 (2) Å] and C—H⋯π inter­actions present.

## Related literature

The title compound may be used as a precursor for obtaining bioactive mol­ecules, see: Otmar *et al.* (2004 ▶). For the biological activity of pyrrolo­pyrimidine derivatives, see: Pudziuvelyte *et al.* (2009 ▶); Kamath *et al.* (2009 ▶). For related structures, see: He *et al.* (2007*a* ▶,*b* ▶).
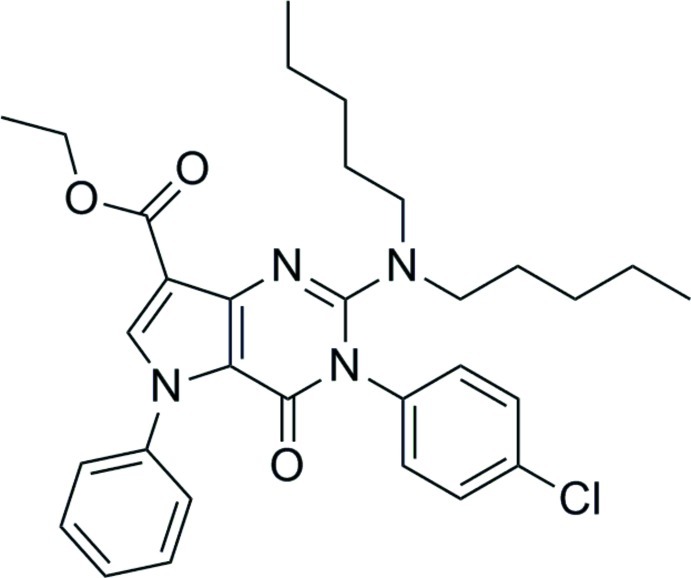


## Experimental

### 

#### Crystal data

C_31_H_37_ClN_4_O_3_
*M* *_r_* = 549.10
Triclinic, 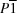

*a* = 9.661 (3) Å
*b* = 12.422 (4) Å
*c* = 14.007 (4) Å
α = 72.110 (5)°
β = 82.697 (6)°
γ = 70.184 (5)°
*V* = 1504.4 (8) Å^3^
*Z* = 2
Mo *K*α radiation
μ = 0.16 mm^−1^
*T* = 298 K
0.30 × 0.10 × 0.10 mm

#### Data collection

Bruker APEXII CCD diffractometer
Absorption correction: multi-scan (*SADABS*; Sheldrick, 1997 ▶) *T*_min_ = 0.943, *T*_max_ = 0.984
9954 measured reflections
5241 independent reflections
3801 reflections with *I* > 2σ(*I*)
*R*_int_ = 0.033

#### Refinement

*R*[*F* ^2^ > 2σ(*F* ^2^)] = 0.054
*wR*(*F* ^2^) = 0.182
*S* = 1.09
5241 reflections
386 parameters
42 restraints
H-atom parameters constrained
Δρ_max_ = 0.35 e Å^−3^
Δρ_min_ = −0.26 e Å^−3^

### 

Data collection: *APEX2* (Bruker, 2001 ▶); cell refinement: *SAINT-Plus* (Bruker, 2001 ▶); data reduction: *SAINT-Plus*; program(s) used to solve structure: *SHELXS97* (Sheldrick, 2008 ▶); program(s) used to refine structure: *SHELXL97* (Sheldrick, 2008 ▶); molecular graphics: *PLATON* (Spek, 2009 ▶); software used to prepare material for publication: *SHELXTL* (Sheldrick, 2008 ▶).

## Supplementary Material

Crystal structure: contains datablock(s) I, global. DOI: 10.1107/S1600536812024609/su2435sup1.cif

Structure factors: contains datablock(s) I. DOI: 10.1107/S1600536812024609/su2435Isup2.hkl

Supplementary material file. DOI: 10.1107/S1600536812024609/su2435Isup3.cml

Additional supplementary materials:  crystallographic information; 3D view; checkCIF report

## Figures and Tables

**Table 1:** Hydrogen-bond geometry (Å, °) *Cg*2 and *Cg*3 are the centroid of the N1,N2,C7–C10 and C1–C6 rings, respectively.

*D*—H⋯*A*	*D*—H	H⋯*A*	*D*⋯*A*	*D*—H⋯*A*
C3—H3⋯O3^i^	0.93	2.57	3.469 (3)	162
C21—H21⋯O3^ii^	0.93	2.52	3.375 (3)	153
C24—H24⋯O1^iii^	0.93	2.58	3.262 (4)	131
C12—H12*A*⋯*Cg*3	0.97	2.77	3.478 (4)	131
C15—H15*A*⋯*Cg*2^iv^	0.96	2.86	3.683 (4)	144

Symmetry codes: (i) 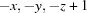; (ii) 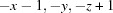; (iii) 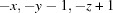; (iv) 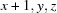.

**Fig. 1.**
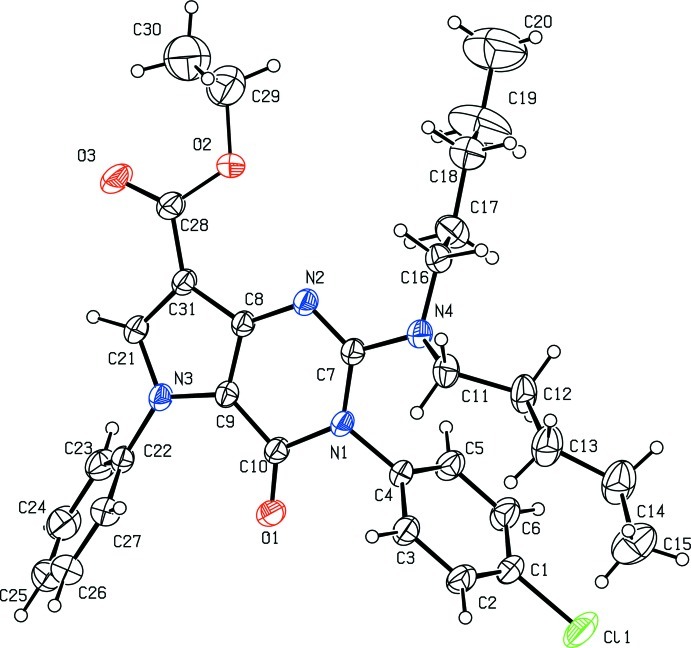
The molecular structure of the title compound, showing the atom-labeling. The displacement ellipsoids are drawn at the 30% probability level. Only the major component of the disordered ethoxy group (atoms O2,C29,C30) are shown.

**Fig. 2.**
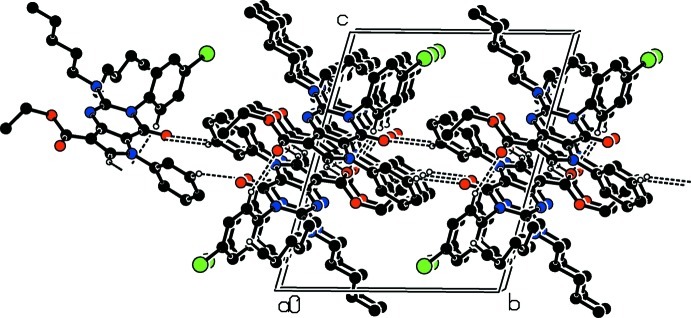
A view along the b axis of the crystal packing in the title compound. The C-H···O hydrogen bonds are shown as dashed lines (see Table 1 for details).

## supplementary crystallographic information

### Comment

The title compound may be used as a precursor for obtaining bioactive molecules (Otmar *et al.*, 2004). The biological activity of pyrrolopyrimidine derivatives, such as anticancer activity and potent purine nucleoside phosphorylase inhibitors, have been described by (Pudziuvelyte *et al.*, 2009 and Kamath *et al.*, 2009). As a part of our ongoing work on the preparation of derivatives of heterocyclic compounds, such as carbonitrile substituted pyrrolo[3,2-d]pyrimidine derivatives (He *et al.*, 2007*a*,*b*), we have synthesized the title compound, and report herein on its crystal structure.

In the title molecule, Fig. 1, the two rings (N3,C8,C9,C31,C21 and N1,N2,C7-C10) of the pyrrolo[3,2-*d*]pyrimidine moiety are nearly coplanar, with a dihedral angle of 5.80 (11)°. The phenyl ring (C22-C27) and the benzene ring (C1-C6) are inclined to the mean plane of the pyrrolo[3,2-*d*]pyrimidine moiety [maximum deviation 0.077 (2) Å for atom C9] by 61.05 (12) and 75.39 (10)°, respectively.

In the crystal, molecules are linked by weak intramolecular C—H···O hydrogen bonds forming a two-dimensional network lying parallel to the ab plane (Table 1 and Fig. 2). There are also C-H···π interactions (Table 1) and π–π interactions present. The latter involve inversion related pyrrolo[3,2-*d*]pyrimidine moieties [Cg1···Cg2^i^ 3.5954 (17) Å; Cg1 centroid of the N3,C8,C9,C21,C31 ring; Cg2 centroid of the N1,N2,C7-C10 ring; symmetry code: (i) -x, -y, -z+1], and inversion related phenyl rings [Cg4···Cg4^ii^ 3.965 (2) Å; perpendicular separation 3.5830 (13) Å, slippage 1.699 Å; Cg4 is the centroid of the C22-C27 ring; symmetry code: (ii) -x, -y-1, -z+1].

### Experimental

To a solution of diethyl 1-phenyl-3- ((triphenylphosphoranylidene)amino)-1H-pyrrole-2,4-dicarboxylate (1.69 g, 3 mmol) in dry methylene dichloride (15 mL) was added 4-chlorophenyl isocyanate (0.46 g, 3 mmol) under nitrogen at room temperature. After the reaction mixture was left to stand for 6 h at 273-278 K, the solvent was removed under reduced pressure and ether/petroleum ether (1:2, 12 mL) was added to precipitate triphenylphosphine oxide. After filtration, dipentylamine (0.47 g, 3 mmol) and anhydrous ethanol (15 mL) were added to the solution. The reaction mixture was allowed to stand for 3 h, then the solvent was removed and anhydrous ethanol (10 ml) with several drops of EtONa in EtOH was added. The mixture was stirred for 1 h at room temperature. The solution was concentrated under reduced pressure and the residue was recrystallized from ethanol to give the title compound. It was recrystallized from ethanol/methylene dichloride (1:1; v:v) at room temperature to give colourless block-like crystals suitable for X-ray diffraction analysis.

### Refinement

C-bound H-atoms were included in calculated positions and treated as riding atoms: C-H = 0.93, 0.97 and 0.96 Å for CH, CH_2_, and CH_3_ H-atoms, respectively, with U_iso_(H) = k × U_eq_(C), where k = 1.5 for CH_3_ H-atoms, and = 1.2 for all other H-atoms. The ethoxy group (atoms O2,C29,C30 & O2',C29',C30') is disordered over two positions; the site occupancies were finally fixed at 0.54 and 0.46.

### Crystal data

**Table:**

C_31_H_37_ClN_4_O_3_	*Z* = 2
*M**_r_* = 549.10	*F*(000) = 584
Triclinic, *P*1	*D*_x_ = 1.212 Mg m^−^^3^
Hall symbol: -P 1	Mo *K*α radiation, λ = 0.71073 Å
*a* = 9.661 (3) Å	Cell parameters from 3181 reflections
*b* = 12.422 (4) Å	θ = 2.4–26.4°
*c* = 14.007 (4) Å	µ = 0.16 mm^−^^1^
α = 72.110 (5)°	*T* = 298 K
β = 82.697 (6)°	Block, colourless
γ = 70.184 (5)°	0.30 × 0.10 × 0.10 mm
*V* = 1504.4 (8) Å^3^	

### Data collection

**Table:**

Bruker APEXII CCD diffractometer	5241 independent reflections
Radiation source: fine-focus sealed tube	3801 reflections with *I* > 2σ(*I*)
Graphite monochromator	*R*_int_ = 0.033
φ and ω scans	θ_max_ = 25.0°, θ_min_ = 1.5°
Absorption correction: multi-scan (*SADABS*; Sheldrick, 1997)	*h* = −11→10
*T*_min_ = 0.943, *T*_max_ = 0.984	*k* = −14→14
9954 measured reflections	*l* = −16→16

### Refinement

**Table:**

Refinement on *F*^2^	Secondary atom site location: difference Fourier map
Least-squares matrix: full	Hydrogen site location: inferred from neighbouring sites
*R*[*F*^2^ > 2σ(*F*^2^)] = 0.054	H-atom parameters constrained
*wR*(*F*^2^) = 0.182	*w* = 1/[σ^2^(*F*_o_^2^) + (0.1048*P*)^2^ + 0.1603*P*] where *P* = (*F*_o_^2^ + 2*F*_c_^2^)/3
*S* = 1.09	(Δ/σ)_max_ = 0.001
5241 reflections	Δρ_max_ = 0.35 e Å^−^^3^
386 parameters	Δρ_min_ = −0.26 e Å^−^^3^
42 restraints	Extinction correction: *SHELXL97* (Sheldrick, 2008), Fc^*^=kFc[1+0.001xFc^2^λ^3^/sin(2θ)]^-1/4^
Primary atom site location: structure-invariant direct methods	Extinction coefficient: 0.029 (4)

### Special details

**Table:**

Geometry. All e.s.d.'s (except the e.s.d. in the dihedral angle between two l.s. planes) are estimated using the full covariance matrix. The cell e.s.d.'s are taken into account individually in the estimation of e.s.d.'s in distances, angles and torsion angles; correlations between e.s.d.'s in cell parameters are only used when they are defined by crystal symmetry. An approximate (isotropic) treatment of cell e.s.d.'s is used for estimating e.s.d.'s involving l.s. planes.
Refinement. Refinement of *F*^2^ against ALL reflections. The weighted *R*-factor *wR* and goodness of fit *S* are based on *F*^2^, conventional *R*-factors *R* are based on *F*, with *F* set to zero for negative *F*^2^. The threshold expression of *F*^2^ > σ(*F*^2^) is used only for calculating *R*-factors(gt) *etc*. and is not relevant to the choice of reflections for refinement. *R*-factors based on *F*^2^ are statistically about twice as large as those based on *F*, and *R*- factors based on ALL data will be even larger.

### Fractional atomic coordinates and isotropic or equivalent isotropic displacement parameters (Å^2^)

**Table:**

	*x*	*y*	*z*	*U*_iso_*/*U*_eq_	Occ. (<1)
C1	0.5649 (3)	−0.2757 (2)	0.17362 (19)	0.0605 (7)	
C2	0.5823 (3)	−0.2786 (2)	0.27016 (19)	0.0621 (7)	
H2	0.6727	−0.3194	0.3008	0.075*	
C3	0.4628 (3)	−0.2197 (2)	0.32125 (18)	0.0535 (6)	
H3	0.4721	−0.2216	0.3871	0.064*	
C4	0.3302 (2)	−0.15846 (19)	0.27457 (16)	0.0427 (5)	
C5	0.3144 (3)	−0.1581 (2)	0.17863 (18)	0.0562 (6)	
H5	0.2240	−0.1178	0.1479	0.067*	
C6	0.4326 (3)	−0.2176 (3)	0.1273 (2)	0.0647 (7)	
H6	0.4223	−0.2181	0.0623	0.078*	
C7	0.1369 (2)	0.02435 (19)	0.29938 (16)	0.0423 (5)	
C8	−0.0692 (2)	0.00511 (18)	0.39051 (15)	0.0381 (5)	
C9	−0.0072 (2)	−0.11672 (18)	0.42804 (15)	0.0390 (5)	
C10	0.1361 (2)	−0.17963 (19)	0.39785 (16)	0.0433 (5)	
C11	0.3656 (3)	0.0775 (2)	0.25637 (19)	0.0551 (6)	
H11A	0.3991	0.0072	0.3128	0.066*	
H11B	0.3620	0.1463	0.2768	0.066*	
C12	0.4759 (3)	0.0666 (3)	0.1695 (2)	0.0673 (7)	
H12A	0.4629	0.0104	0.1390	0.081*	
H12B	0.4550	0.1435	0.1192	0.081*	
C13	0.6346 (3)	0.0259 (3)	0.1994 (2)	0.0791 (9)	
H13A	0.6489	−0.0405	0.2600	0.095*	
H13B	0.6529	0.0905	0.2154	0.095*	
C14	0.7479 (3)	−0.0127 (4)	0.1196 (3)	0.0934 (11)	
H14A	0.7158	−0.0627	0.0920	0.112*	
H14B	0.7497	0.0578	0.0655	0.112*	
C15	0.8970 (4)	−0.0774 (4)	0.1548 (3)	0.1099 (13)	
H15A	0.9267	−0.0326	0.1886	0.165*	
H15B	0.9627	−0.0886	0.0986	0.165*	
H15C	0.9000	−0.1537	0.2004	0.165*	
C16	0.1306 (3)	0.2113 (2)	0.1768 (2)	0.0593 (7)	
H16A	0.1981	0.2542	0.1420	0.071*	
H16B	0.0733	0.2519	0.2249	0.071*	
C17	0.0288 (4)	0.2168 (3)	0.1022 (2)	0.0765 (8)	
H17A	−0.0349	0.1701	0.1364	0.092*	
H17B	0.0868	0.1799	0.0520	0.092*	
C18	−0.0650 (4)	0.3403 (3)	0.0502 (3)	0.0929 (11)	
H18A	−0.0011	0.3863	0.0149	0.111*	
H18B	−0.1211	0.3776	0.1006	0.111*	
C19	−0.1715 (7)	0.3471 (4)	−0.0243 (4)	0.157 (2)	
H19A	−0.1143	0.3089	−0.0740	0.189*	
H19B	−0.2340	0.3000	0.0115	0.189*	
C20	−0.2658 (8)	0.4638 (5)	−0.0773 (5)	0.187 (3)	
H20A	−0.3324	0.4994	−0.0303	0.281*	
H20B	−0.3209	0.4558	−0.1257	0.281*	
H20C	−0.2067	0.5134	−0.1110	0.281*	
C21	−0.2389 (2)	−0.07124 (19)	0.48391 (16)	0.0441 (5)	
H21	−0.3270	−0.0786	0.5160	0.053*	
C22	−0.0964 (2)	−0.28502 (19)	0.53985 (16)	0.0436 (5)	
C23	−0.1896 (3)	−0.3390 (2)	0.5213 (2)	0.0607 (7)	
H23	−0.2614	−0.2964	0.4732	0.073*	
C24	−0.1771 (4)	−0.4547 (3)	0.5732 (3)	0.0774 (9)	
H24	−0.2385	−0.4917	0.5598	0.093*	
C25	−0.0728 (4)	−0.5153 (3)	0.6453 (3)	0.0847 (11)	
H25	−0.0638	−0.5939	0.6813	0.102*	
C26	0.0186 (4)	−0.4612 (3)	0.6650 (2)	0.0757 (8)	
H26	0.0882	−0.5031	0.7146	0.091*	
C27	0.0080 (3)	−0.3454 (2)	0.61195 (18)	0.0564 (6)	
H27	0.0705	−0.3089	0.6247	0.068*	
C28	−0.3371 (3)	0.1472 (2)	0.41352 (18)	0.0513 (6)	
O2	−0.3074 (8)	0.2389 (6)	0.3374 (4)	0.0579 (16)	0.54
C29	−0.4238 (12)	0.3582 (9)	0.3102 (8)	0.106 (3)	0.54
H29A	−0.5180	0.3480	0.3066	0.127*	0.54
H29B	−0.4004	0.4054	0.2448	0.127*	0.54
C30	−0.4335 (10)	0.4226 (8)	0.3879 (7)	0.128 (3)	0.54
H30A	−0.4741	0.3836	0.4497	0.193*	0.54
H30B	−0.4957	0.5038	0.3643	0.193*	0.54
H30C	−0.3369	0.4213	0.3991	0.193*	0.54
O2'	−0.2896 (9)	0.2395 (7)	0.3724 (5)	0.0584 (18)	0.46
C29'	−0.4027 (9)	0.3551 (6)	0.3531 (7)	0.070 (2)	0.46
H29C	−0.3579	0.4150	0.3506	0.084*	0.46
H29D	−0.4738	0.3540	0.4087	0.084*	0.46
C30'	−0.4826 (14)	0.3907 (12)	0.2571 (9)	0.147 (5)	0.46
H30D	−0.4127	0.3869	0.2021	0.220*	0.46
H30E	−0.5492	0.4707	0.2461	0.220*	0.46
H30F	−0.5371	0.3373	0.2620	0.220*	0.46
C31	−0.2180 (2)	0.03478 (19)	0.42781 (16)	0.0411 (5)	
Cl1	0.71506 (9)	−0.34780 (9)	0.10790 (6)	0.1034 (4)	
N1	0.20328 (18)	−0.09956 (15)	0.32853 (13)	0.0417 (4)	
N2	0.00551 (19)	0.07781 (15)	0.32903 (13)	0.0431 (4)	
N3	−0.11308 (19)	−0.16311 (15)	0.48561 (13)	0.0413 (4)	
N4	0.2161 (2)	0.09019 (17)	0.23112 (15)	0.0512 (5)	
O1	0.19808 (19)	−0.28622 (14)	0.41939 (15)	0.0648 (5)	
O3	−0.45802 (18)	0.15757 (16)	0.45300 (16)	0.0703 (5)	

### Atomic displacement parameters (Å^2^)

**Table:**

	*U*^11^	*U*^22^	*U*^33^	*U*^12^	*U*^13^	*U*^23^
C1	0.0476 (14)	0.0637 (15)	0.0541 (15)	−0.0005 (12)	0.0113 (12)	−0.0183 (12)
C2	0.0411 (13)	0.0717 (17)	0.0558 (15)	0.0019 (12)	−0.0012 (11)	−0.0154 (13)
C3	0.0415 (13)	0.0670 (15)	0.0436 (13)	−0.0068 (11)	0.0010 (10)	−0.0163 (11)
C4	0.0345 (11)	0.0458 (12)	0.0445 (12)	−0.0110 (9)	0.0078 (9)	−0.0135 (9)
C5	0.0399 (13)	0.0698 (16)	0.0522 (14)	−0.0059 (12)	−0.0021 (11)	−0.0203 (12)
C6	0.0595 (16)	0.0802 (18)	0.0487 (14)	−0.0088 (14)	0.0040 (12)	−0.0276 (13)
C7	0.0392 (12)	0.0435 (12)	0.0435 (12)	−0.0148 (10)	0.0068 (9)	−0.0126 (10)
C8	0.0326 (11)	0.0432 (11)	0.0381 (11)	−0.0124 (9)	0.0029 (9)	−0.0120 (9)
C9	0.0357 (11)	0.0428 (11)	0.0403 (11)	−0.0149 (9)	0.0054 (9)	−0.0141 (9)
C10	0.0375 (12)	0.0421 (12)	0.0500 (13)	−0.0132 (10)	0.0081 (10)	−0.0158 (10)
C11	0.0529 (14)	0.0643 (15)	0.0544 (14)	−0.0298 (12)	0.0105 (11)	−0.0178 (12)
C12	0.0478 (15)	0.098 (2)	0.0561 (15)	−0.0347 (15)	0.0102 (12)	−0.0143 (14)
C13	0.0550 (17)	0.114 (3)	0.0741 (19)	−0.0374 (17)	0.0087 (14)	−0.0274 (18)
C14	0.0560 (19)	0.139 (3)	0.086 (2)	−0.035 (2)	0.0066 (16)	−0.032 (2)
C15	0.067 (2)	0.131 (3)	0.130 (3)	−0.013 (2)	−0.009 (2)	−0.052 (3)
C16	0.0582 (15)	0.0456 (13)	0.0661 (16)	−0.0190 (11)	0.0189 (13)	−0.0103 (12)
C17	0.093 (2)	0.0589 (16)	0.0670 (18)	−0.0211 (16)	0.0017 (16)	−0.0077 (14)
C18	0.084 (2)	0.076 (2)	0.089 (2)	−0.0084 (18)	0.0040 (19)	−0.0038 (18)
C19	0.205 (6)	0.094 (3)	0.153 (4)	−0.018 (3)	−0.092 (5)	−0.007 (3)
C20	0.201 (7)	0.140 (5)	0.179 (6)	0.001 (5)	−0.075 (5)	−0.021 (4)
C21	0.0331 (11)	0.0511 (13)	0.0473 (12)	−0.0131 (10)	0.0070 (9)	−0.0164 (10)
C22	0.0409 (12)	0.0422 (12)	0.0459 (12)	−0.0146 (10)	0.0126 (10)	−0.0137 (10)
C23	0.0484 (14)	0.0561 (15)	0.0821 (18)	−0.0209 (12)	0.0093 (13)	−0.0254 (14)
C24	0.0684 (19)	0.0572 (17)	0.113 (3)	−0.0324 (15)	0.0261 (19)	−0.0305 (18)
C25	0.095 (3)	0.0448 (15)	0.098 (2)	−0.0232 (17)	0.041 (2)	−0.0140 (16)
C26	0.077 (2)	0.0582 (16)	0.0685 (18)	−0.0071 (15)	0.0071 (15)	−0.0047 (14)
C27	0.0552 (15)	0.0511 (14)	0.0564 (15)	−0.0133 (12)	0.0000 (12)	−0.0108 (12)
C28	0.0393 (13)	0.0509 (13)	0.0595 (14)	−0.0110 (10)	0.0029 (11)	−0.0154 (11)
O2	0.048 (2)	0.045 (2)	0.057 (3)	0.0009 (17)	0.004 (2)	0.000 (2)
C29	0.099 (5)	0.114 (5)	0.090 (5)	−0.017 (4)	0.005 (4)	−0.032 (4)
C30	0.113 (5)	0.118 (4)	0.155 (5)	−0.047 (4)	0.000 (4)	−0.030 (4)
O2'	0.050 (3)	0.047 (3)	0.067 (4)	−0.009 (2)	−0.001 (3)	−0.009 (3)
C29'	0.070 (4)	0.045 (3)	0.083 (4)	−0.003 (3)	0.004 (3)	−0.020 (3)
C30'	0.136 (6)	0.146 (6)	0.138 (6)	−0.034 (4)	−0.012 (4)	−0.020 (4)
C31	0.0333 (11)	0.0461 (12)	0.0425 (12)	−0.0104 (9)	0.0030 (9)	−0.0145 (10)
Cl1	0.0746 (6)	0.1193 (7)	0.0770 (6)	0.0211 (5)	0.0203 (4)	−0.0398 (5)
N1	0.0322 (9)	0.0423 (10)	0.0477 (10)	−0.0110 (8)	0.0104 (8)	−0.0143 (8)
N2	0.0383 (10)	0.0431 (10)	0.0475 (10)	−0.0141 (8)	0.0073 (8)	−0.0144 (8)
N3	0.0349 (10)	0.0448 (10)	0.0446 (10)	−0.0155 (8)	0.0069 (8)	−0.0130 (8)
N4	0.0424 (11)	0.0473 (11)	0.0584 (12)	−0.0172 (9)	0.0126 (9)	−0.0096 (9)
O1	0.0516 (10)	0.0408 (9)	0.0872 (13)	−0.0099 (8)	0.0225 (9)	−0.0123 (8)
O3	0.0378 (10)	0.0626 (11)	0.0981 (14)	−0.0077 (8)	0.0183 (9)	−0.0224 (10)

### Geometric parameters (Å, º)

**Table:**

C1—C6	1.369 (4)	C17—H17B	0.9700
C1—C2	1.372 (4)	C18—C19	1.520 (6)
C1—Cl1	1.743 (2)	C18—H18A	0.9700
C2—C3	1.386 (3)	C18—H18B	0.9700
C2—H2	0.9300	C19—C20	1.448 (6)
C3—C4	1.376 (3)	C19—H19A	0.9700
C3—H3	0.9300	C19—H19B	0.9700
C4—C5	1.370 (3)	C20—H20A	0.9600
C4—N1	1.451 (3)	C20—H20B	0.9600
C5—C6	1.382 (3)	C20—H20C	0.9600
C5—H5	0.9300	C21—N3	1.353 (3)
C6—H6	0.9300	C21—C31	1.377 (3)
C7—N2	1.300 (3)	C21—H21	0.9300
C7—N4	1.388 (3)	C22—C27	1.372 (3)
C7—N1	1.399 (3)	C22—C23	1.380 (3)
C8—N2	1.373 (3)	C22—N3	1.434 (3)
C8—C9	1.379 (3)	C23—C24	1.368 (4)
C8—C31	1.429 (3)	C23—H23	0.9300
C9—N3	1.390 (3)	C24—C25	1.371 (5)
C9—C10	1.423 (3)	C24—H24	0.9300
C10—O1	1.211 (3)	C25—C26	1.374 (5)
C10—N1	1.428 (3)	C25—H25	0.9300
C11—N4	1.476 (3)	C26—C27	1.376 (4)
C11—C12	1.519 (3)	C26—H26	0.9300
C11—H11A	0.9700	C27—H27	0.9300
C11—H11B	0.9700	C28—O3	1.210 (3)
C12—C13	1.513 (4)	C28—O2'	1.319 (9)
C12—H12A	0.9700	C28—O2	1.383 (7)
C12—H12B	0.9700	C28—C31	1.453 (3)
C13—C14	1.531 (4)	O2—C29	1.496 (10)
C13—H13A	0.9700	C29—C30	1.514 (8)
C13—H13B	0.9700	C29—H29A	0.9700
C14—C15	1.454 (5)	C29—H29B	0.9700
C14—H14A	0.9700	C30—H30A	0.9600
C14—H14B	0.9700	C30—H30B	0.9600
C15—H15A	0.9600	C30—H30C	0.9600
C15—H15B	0.9600	O2'—C29'	1.450 (9)
C15—H15C	0.9600	C29'—C30'	1.505 (9)
C16—N4	1.463 (3)	C29'—H29C	0.9700
C16—C17	1.495 (4)	C29'—H29D	0.9700
C16—H16A	0.9700	C30'—H30D	0.9600
C16—H16B	0.9700	C30'—H30E	0.9600
C17—C18	1.499 (4)	C30'—H30F	0.9600
C17—H17A	0.9700		
			
C6—C1—C2	121.7 (2)	C17—C18—H18B	108.5
C6—C1—Cl1	119.0 (2)	C19—C18—H18B	108.5
C2—C1—Cl1	119.3 (2)	H18A—C18—H18B	107.5
C1—C2—C3	118.8 (2)	C20—C19—C18	118.1 (4)
C1—C2—H2	120.6	C20—C19—H19A	107.8
C3—C2—H2	120.6	C18—C19—H19A	107.8
C4—C3—C2	120.0 (2)	C20—C19—H19B	107.8
C4—C3—H3	120.0	C18—C19—H19B	107.8
C2—C3—H3	120.0	H19A—C19—H19B	107.1
C5—C4—C3	120.3 (2)	C19—C20—H20A	109.5
C5—C4—N1	119.3 (2)	C19—C20—H20B	109.5
C3—C4—N1	120.31 (19)	H20A—C20—H20B	109.5
C4—C5—C6	120.2 (2)	C19—C20—H20C	109.5
C4—C5—H5	119.9	H20A—C20—H20C	109.5
C6—C5—H5	119.9	H20B—C20—H20C	109.5
C1—C6—C5	119.0 (2)	N3—C21—C31	110.33 (19)
C1—C6—H6	120.5	N3—C21—H21	124.8
C5—C6—H6	120.5	C31—C21—H21	124.8
N2—C7—N4	119.96 (19)	C27—C22—C23	120.7 (2)
N2—C7—N1	123.54 (19)	C27—C22—N3	119.9 (2)
N4—C7—N1	116.42 (18)	C23—C22—N3	119.4 (2)
N2—C8—C9	123.51 (18)	C24—C23—C22	120.4 (3)
N2—C8—C31	129.65 (19)	C24—C23—H23	119.8
C9—C8—C31	106.85 (18)	C22—C23—H23	119.8
C8—C9—N3	108.75 (18)	C23—C24—C25	119.1 (3)
C8—C9—C10	122.38 (19)	C23—C24—H24	120.5
N3—C9—C10	128.34 (19)	C25—C24—H24	120.5
O1—C10—C9	128.7 (2)	C24—C25—C26	120.6 (3)
O1—C10—N1	120.30 (19)	C24—C25—H25	119.7
C9—C10—N1	110.90 (18)	C26—C25—H25	119.7
N4—C11—C12	113.0 (2)	C25—C26—C27	120.5 (3)
N4—C11—H11A	109.0	C25—C26—H26	119.7
C12—C11—H11A	109.0	C27—C26—H26	119.7
N4—C11—H11B	109.0	C22—C27—C26	118.7 (3)
C12—C11—H11B	109.0	C22—C27—H27	120.7
H11A—C11—H11B	107.8	C26—C27—H27	120.7
C13—C12—C11	113.8 (2)	O3—C28—O2'	122.2 (4)
C13—C12—H12A	108.8	O3—C28—O2	122.0 (3)
C11—C12—H12A	108.8	O2'—C28—O2	23.0 (4)
C13—C12—H12B	108.8	O3—C28—C31	124.2 (2)
C11—C12—H12B	108.8	O2'—C28—C31	111.8 (4)
H12A—C12—H12B	107.7	O2—C28—C31	113.0 (3)
C12—C13—C14	114.7 (3)	C28—O2—C29	118.9 (7)
C12—C13—H13A	108.6	O2—C29—C30	110.0 (8)
C14—C13—H13A	108.6	O2—C29—H29A	109.7
C12—C13—H13B	108.6	C30—C29—H29A	109.7
C14—C13—H13B	108.6	O2—C29—H29B	109.7
H13A—C13—H13B	107.6	C30—C29—H29B	109.7
C15—C14—C13	115.1 (3)	H29A—C29—H29B	108.2
C15—C14—H14A	108.5	C28—O2'—C29'	115.2 (7)
C13—C14—H14A	108.5	O2'—C29'—C30'	114.0 (8)
C15—C14—H14B	108.5	O2'—C29'—H29C	108.8
C13—C14—H14B	108.5	C30'—C29'—H29C	108.8
H14A—C14—H14B	107.5	O2'—C29'—H29D	108.8
C14—C15—H15A	109.5	C30'—C29'—H29D	108.8
C14—C15—H15B	109.5	H29C—C29'—H29D	107.6
H15A—C15—H15B	109.5	C29'—C30'—H30D	109.5
C14—C15—H15C	109.5	C29'—C30'—H30E	109.5
H15A—C15—H15C	109.5	H30D—C30'—H30E	109.5
H15B—C15—H15C	109.5	C29'—C30'—H30F	109.5
N4—C16—C17	114.1 (2)	H30D—C30'—H30F	109.5
N4—C16—H16A	108.7	H30E—C30'—H30F	109.5
C17—C16—H16A	108.7	C21—C31—C8	106.30 (18)
N4—C16—H16B	108.7	C21—C31—C28	121.2 (2)
C17—C16—H16B	108.7	C8—C31—C28	132.4 (2)
H16A—C16—H16B	107.6	C7—N1—C10	123.25 (17)
C16—C17—C18	114.5 (3)	C7—N1—C4	121.88 (17)
C16—C17—H17A	108.6	C10—N1—C4	113.57 (16)
C18—C17—H17A	108.6	C7—N2—C8	116.07 (18)
C16—C17—H17B	108.6	C21—N3—C9	107.75 (17)
C18—C17—H17B	108.6	C21—N3—C22	124.40 (17)
H17A—C17—H17B	107.6	C9—N3—C22	127.83 (17)
C17—C18—C19	115.1 (3)	C7—N4—C16	115.76 (19)
C17—C18—H18A	108.5	C7—N4—C11	118.94 (19)
C19—C18—H18A	108.5	C16—N4—C11	114.48 (19)
			
C6—C1—C2—C3	−0.8 (4)	C9—C8—C31—C21	−1.4 (2)
Cl1—C1—C2—C3	179.2 (2)	N2—C8—C31—C28	0.6 (4)
C1—C2—C3—C4	−0.8 (4)	C9—C8—C31—C28	−179.2 (2)
C2—C3—C4—C5	1.9 (4)	O3—C28—C31—C21	4.3 (4)
C2—C3—C4—N1	178.1 (2)	O2'—C28—C31—C21	169.4 (3)
C3—C4—C5—C6	−1.2 (4)	O2—C28—C31—C21	−165.7 (3)
N1—C4—C5—C6	−177.5 (2)	O3—C28—C31—C8	−178.3 (2)
C2—C1—C6—C5	1.5 (4)	O2'—C28—C31—C8	−13.1 (5)
Cl1—C1—C6—C5	−178.5 (2)	O2—C28—C31—C8	11.8 (4)
C4—C5—C6—C1	−0.4 (4)	N2—C7—N1—C10	−3.2 (3)
N2—C8—C9—N3	−178.86 (18)	N4—C7—N1—C10	−179.81 (19)
C31—C8—C9—N3	1.0 (2)	N2—C7—N1—C4	162.9 (2)
N2—C8—C9—C10	−6.6 (3)	N4—C7—N1—C4	−13.7 (3)
C31—C8—C9—C10	173.22 (19)	O1—C10—N1—C7	179.2 (2)
C8—C9—C10—O1	−174.2 (2)	C9—C10—N1—C7	2.8 (3)
N3—C9—C10—O1	−3.5 (4)	O1—C10—N1—C4	12.0 (3)
C8—C9—C10—N1	1.8 (3)	C9—C10—N1—C4	−164.37 (17)
N3—C9—C10—N1	172.48 (19)	C5—C4—N1—C7	−67.3 (3)
N4—C11—C12—C13	−166.9 (2)	C3—C4—N1—C7	116.4 (2)
C11—C12—C13—C14	166.9 (3)	C5—C4—N1—C10	100.1 (2)
C12—C13—C14—C15	−166.3 (3)	C3—C4—N1—C10	−76.2 (3)
N4—C16—C17—C18	176.9 (2)	N4—C7—N2—C8	175.16 (18)
C16—C17—C18—C19	−178.8 (4)	N1—C7—N2—C8	−1.4 (3)
C17—C18—C19—C20	179.9 (5)	C9—C8—N2—C7	6.2 (3)
C27—C22—C23—C24	−1.4 (4)	C31—C8—N2—C7	−173.6 (2)
N3—C22—C23—C24	−179.0 (2)	C31—C21—N3—C9	−0.9 (2)
C22—C23—C24—C25	1.3 (4)	C31—C21—N3—C22	178.10 (19)
C23—C24—C25—C26	−0.3 (5)	C8—C9—N3—C21	−0.1 (2)
C24—C25—C26—C27	−0.7 (5)	C10—C9—N3—C21	−171.8 (2)
C23—C22—C27—C26	0.4 (4)	C8—C9—N3—C22	−179.00 (19)
N3—C22—C27—C26	178.0 (2)	C10—C9—N3—C22	9.3 (3)
C25—C26—C27—C22	0.7 (4)	C27—C22—N3—C21	−121.0 (2)
O3—C28—O2—C29	6.3 (8)	C23—C22—N3—C21	56.6 (3)
O2'—C28—O2—C29	−91.7 (15)	C27—C22—N3—C9	57.7 (3)
C31—C28—O2—C29	176.5 (6)	C23—C22—N3—C9	−124.6 (2)
C28—O2—C29—C30	76.2 (10)	N2—C7—N4—C16	−16.0 (3)
O3—C28—O2'—C29'	−16.2 (8)	N1—C7—N4—C16	160.8 (2)
O2—C28—O2'—C29'	80.6 (15)	N2—C7—N4—C11	126.5 (2)
C31—C28—O2'—C29'	178.3 (5)	N1—C7—N4—C11	−56.8 (3)
C28—O2'—C29'—C30'	−82.2 (10)	C17—C16—N4—C7	−69.9 (3)
N3—C21—C31—C8	1.4 (2)	C17—C16—N4—C11	146.0 (2)
N3—C21—C31—C28	179.49 (19)	C12—C11—N4—C7	135.7 (2)
N2—C8—C31—C21	178.4 (2)	C12—C11—N4—C16	−81.4 (3)

### Hydrogen-bond geometry (Å, º)

Cg2 and Cg3 are the centroid of the N1,N2,C7–C10 and C1–C6 rings, respectively.

**Table:**

*D*—H···*A*	*D*—H	H···*A*	*D*···*A*	*D*—H···*A*
C3—H3···O3^i^	0.93	2.57	3.469 (3)	162
C21—H21···O3^ii^	0.93	2.52	3.375 (3)	153
C24—H24···O1^iii^	0.93	2.58	3.262 (4)	131
C12—H12*A*···*Cg*3	0.97	2.77	3.478 (4)	131
C15—H15*A*···*Cg*2^iv^	0.96	2.86	3.683 (4)	144

Symmetry codes: (i) −*x*, −*y*, −*z*+1; (ii) −*x*−1, −*y*, −*z*+1; (iii) −*x*, −*y*−1, −*z*+1; (iv) *x*+1, *y*, *z*.

## References

[bb1] Bruker (2001). *SMART* and *SAINT-Plus* Bruker AXS Inc., Madison, Wisconsin, USA.

[bb2] He, P., Peng, X.-M. & Li, G.-H. (2007*a*). *Acta Cryst.* E**63**, o4884.

[bb3] He, P., Zheng, A., Cai, C.-Q. & Fang, C.-L. (2007*b*). *Acta Cryst.* E**63**, o3185.

[bb4] Kamath, V. P., Juarez-Brambila, J. J., Morris, C. B., Winslow, C. D. & Morris, P. E. (2009). *Org. Process Res. Dev.* **13**, 928–932.

[bb5] Otmar, M., Masojidkova, M., Votruba, I. & Holy, A. (2004). *Bioorg. Med. Chem.* **12**, 3187–3195. 10.1016/j.bmc.2004.04.003 15158786

[bb6] Pudziuvelyte, E., Rios-Luci, C., Leon, L. G., Cikotiene, I. & Padron, J. M. (2009). *Bioorg. Med. Chem.* **17**, 4955–4960. 10.1016/j.bmc.2009.05.078 19527934

[bb7] Sheldrick, G. M. (1997). *SADABS* University of Göttingen, Germany.

[bb8] Sheldrick, G. M. (2008). *Acta Cryst.* A**64**, 112–122. 10.1107/S0108767307043930 18156677

[bb9] Spek, A. L. (2009). *Acta Cryst.* D**65**, 148–155. 10.1107/S090744490804362X PMC2631630 19171970

